# Twelve months of exercise training did not halt abdominal aortic calcification in patients with CKD – a sub-study of RENEXC-a randomized controlled trial

**DOI:** 10.1186/s12882-020-01881-y

**Published:** 2020-06-22

**Authors:** Yunan Zhou, Matthias Hellberg, Thomas Hellmark, Peter Höglund, Naomi Clyne

**Affiliations:** 1 Department of Clinical Sciences Lund, Nephrology, Lund, Sweden, Lund University, Skåne University Hospital, Alwallhuset Barngatan 2A, 121 85 Lund, Sweden; 2 Department of Laboratory Medicine, Division of Clinical Chemistry & Pharmacology, Lund University, Skåne University Hospital, Lund, Sweden

**Keywords:** Abdominal aortic calcification, Exercise training, CKD, Lipids, Arteriosclerosis

## Abstract

**Background:**

Arteriosclerosis is prevalent in patients with chronic kidney disease (CKD). Our aims were to investigate (1) the effects of 12 months of either balance- or strength- both in combination with endurance training on abdominal aortic calcification (AAC); on some lipids and calcific- and inflammatory markers; and (2) the relationships between the change in AAC score and these markers in non-dialysis dependent patients with CKD stages 3 to 5.

**Methods:**

One hundred twelve patients (mean age 67 ± 13 years), who completed 12 months of exercise training; comprising either balance- or strength training, both in combination with endurance training; with a measured glomerular filtration rate (mGFR) 22.6 ± 8 mL/min/1.73m^2^, were included in this study. AAC was evaluated with lateral lumbar X-ray using the scoring system described by Kauppila. Plasma fetuin-A, fibroblast growth factor 23 (FGF23) and interleukin 6 (IL6) were measured with Enzyme-linked immunosorbent assay (ELISA) kits.

**Results:**

After 12 months of exercise training, the AAC score increased significantly in both groups; mGFR and lipoprotein (a) decreased significantly in both groups; parathyroid hormone (PTH) and 1,25(OH)_2_D_3_ increased significantly only in the strength group; fetuin-A increased significantly only in the balance group. Plasma triglycerides, total cholesterol, high-density lipoprotein cholesterol, low-density lipoprotein cholesterol, FGF23, phosphate, calcium, IL6, C-reactive protein (CRP), albumin were unchanged. The increase in AAC score was positively related to ageing and the levels of baseline triglycerides and lipoprotein (a).

**Conclusions:**

Exercise training did not prevent the progression of AAC; it might have contributed to the reduced levels of lipoprotein (a) and unchanged levels of calcific- and inflammatory markers in these patients with non-dialysis dependent CKD. Hypertriglyceridemia, high levels of lipoprotein (a) and ageing emerged as longitudinal predictors of vascular calcification in these patients.

**Trial registration:**

NCT02041156 at www.ClinicalTrials.gov. Date of registration: January 20, 2014. Retrospectively registered.

## Background

Cardiovascular disease is the main cause of morbidity and mortality in patients with chronic kidney disease (CKD) [1]. Vascular disease in CKD involves both intimal and medial layers of the arterial wall. The damage of the intimal layer involves endothelial dysfunction and lipid metabolism disorder and results in atherosclerosis [2]. The damage of the medial layer involves mineral metabolism disturbances and results in vascular calcification [2]. Moreover, the two types of vascular disease share some common etiologies, like inflammation, uremic toxins and oxidative stress [2–4]. Dyslipidemia is common in patients with CKD [5] and has been shown to be a risk factor of atherosclerosis and to contribute to the intimal lesion of arteries [6, 7].

Earlier studies have shown that there is a complex interplay between fibroblast growth factor 23 (FGF23), klotho and vitamin D, which affects the regulation of phosphate homeostasis and vascular calcification [3]. Some mineralization inhibitors, like matrix Gla protein (MGP) and fetuin-A, are also involved in this process [4, 8]. Low-grade systemic inflammation, characterized by elevated levels of circulating inflammatory markers like C-reactive protein (CRP) and interleukin 6 (IL6), is frequently observed in patients with CKD. The elevated levels of these inflammatory markers are both a stimulus for and a result of vascular calcification [4].

There are a number of different methods to evaluate vascular calcification. Abdominal aortic calcification (AAC) has been suggested as a useful tool to assess vascular calcification in patients with CKD in the 2009 Kidney Disease Improvement Global Outcomes (KDIGO) clinical practice guideline on CKD mineral and bone disorder [9].

Exercise training has been shown to reduce inflammation [10] and improve vascular endothelial function and arterial stiffness/compliance in patients with CKD [11]. To our knowledge, there are few studies, if any, using the AAC score to follow up the effects of exercise training on vascular calcification in patients with CKD.

In this pre-specified sub-study of RENal EXerCise (RENEXC), our aims were to investigate (1) the effects of 12 months of either balance- or strength- both in combination with endurance training on AAC score; some lipids and calcific- and inflammatory markers; and (2) the relationships between the change in AAC score and these markers in non-dialysis dependent patients with CKD stages 3–5.

## Methods

### Study design

This is a pre-specified sub-study of the RENEXC trial, a randomized controlled, parallel group, interventional, single-center trial with 2 treatment arms with a 1:1 allocation ratio. The included patients all had CKD and were not on renal replacement therapy. RENEXC is registered as NCT02041156 at www.ClinicalTrials.gov, approved by the Regional Ethical Review Board in Lund (Ref 2011/369) and adheres to the Helsinki declaration. Both incident and prevalent patients treated at the Department of Nephrology in Lund, Skåne University Hospital, were included provided that they had an estimated GFR (eGFR) < 30 mL/min/1.73m^2^ prior to inclusion, were adults (age ≥ 18 years). All renal diagnoses and any number of comorbidities were accepted. Patients were excluded if they had an orthopedic impediment, severe neurological dysfunction, inability to understand the patient information, renal replacement therapy and estimated start of dialysis within 12 months of study start. Complete study design and primary data analysis of RENEXC have been presented previously [12]. Some information on study design and methods is repeated here for clarity.

### Randomization and blinding

Random allocation was generated by investigator (PH) with program SAS ProcPlan. All the recruitment staff except the research physiotherapist were blinded to the randomization. The specific randomization method has been published in the primary study [12].

### Intervention and assessment of physical performance

One hundred fifty-one patients were randomly assigned to either balance- or strength training both in combination with endurance training for a total of 150 min per week for 12 months. The 150 min total training time included 60 min of endurance training and 90 min of either balance- or strength training. Training intensity was evaluated by the Rating of Perceived Exertion using the Borg scale and kept within prespecified limits [12, 13]. Details of the intervention and tests used to assess physical performance have been presented previously in the primary study report [12].

The primary outcomes were the measures of physical performance at baseline and after 12 months [12]. The pre-specified secondary outcomes were AAC score and some markers of arteriosclerosis at baseline and after 12 months as well as the relationships between AAC score and these markers. There were no changes to trial outcomes after the trial had commenced.

### Assessment of the AAC score

AAC was evaluated by lateral lumbar X-ray, at the Department of Diagnostic Radiology, Skåne University Hospital, which is accredited by the Swedish Board for Accreditation and Conformity Assessment (SWEDAC), (ISO 15189:2012). The AAC score was calculated using Kauppila’s scoring system [14]. Calcific deposits were graded on a scale of 0–3 on both posterior and anterior sides of each segment: 0 = no calcific deposits, 1 = calcific deposits filling less than 1/3 of the aortic wall, 2 = 1/3 to 2/3 of the aortic wall calcified, 3 = more than 2/3 of the aortic wall calcified. The grades of both posterior and anterior of four segments (Lumbar 1- Lumbar 4) were summed, ranging from 0 to 24. 0 score is normal, 1–6 score is moderate calcification, 7 and above is severe calcification [14, 15]. The grading was performed by one investigator (YZ).

### Measured GFR (mGFR), plasma 1, 25(OH)_2_D_3_ and laboratory analyses

mGFR, plasma 1,25(OH)_2_D_3_ and routine laboratory analyses were analyzed at the Department of Clinical Chemistry, Laboratory Medicine Skåne, which is accredited by SWEDAC (ISO 15189:2012). mGFR was assessed with iohexol clearance [16]. Plasma 1,25(OH)_2_D_3_ was analyzed using liquid chromatography–mass spectrometry.

### Plasma fetuin-A, FGF23 and IL6

Plasma fetuin-A, FGF23 and IL6 were all measured using ELISA kits (R&D systems, Inc., Minneapolis, USA) at the Nephrology Laboratory, Biomedicine Centre at Lund University. The plasma was collected fasting and stored at − 80 °C. Since there are no standard reference ranges for these variables, normal levels were defined as the values in healthy subjects presented in previous studies [17–19].

### Statistical analysis

Descriptive statistics are presented as mean ± SD and/or median with interquartile range. Categorical variables were used to describe frequency and continuous variables for measurements. Paired t-test was used to compare parametric variables, and Wilcoxon signed rank test was used to compare nonparametric variables. Multiple linear regression analysis was performed to analyze the relationships between variables. In a previous study, analyzing the primary outcomes of RENEXC with mixed model analyses, we did not find any statistically significant differences between groups for any measures of physical performance after 12 months of exercise training. Therefore, in this study, we pooled the patients from both groups to increase the power of the multiple linear regression analyses. The level of significance was set at *p* < 0.05**.** Data were analyzed using R software (R foundation for Statistical Computing, Vienna, Austria).

## Results

**Fig. 1:**
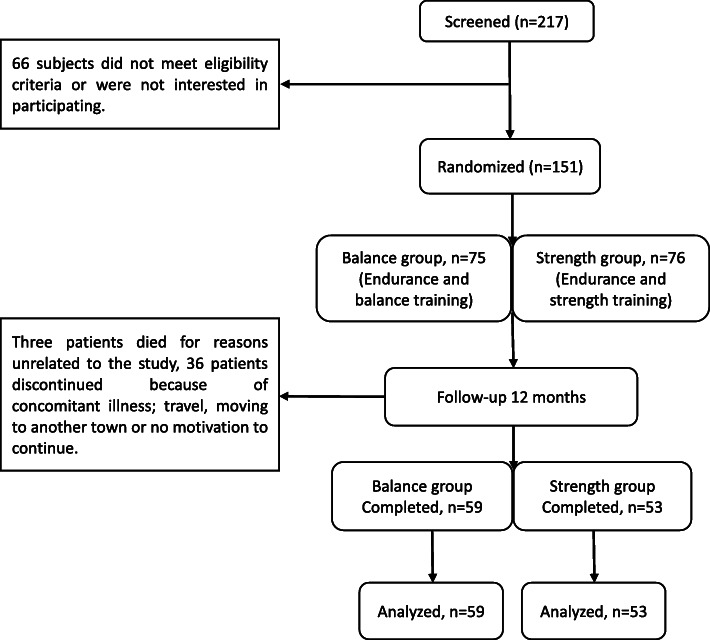
CONSORT flow of 12 months

Two hundred seventeen patients were screened of whom 151 patients were randomized and 112 patients completed 12 months of exercise training. The 112 completers (mean age 67 ± 13 years, average mGFR 22.6 ± 8 mL/min/1.73m^2^) were included in this study. Two patients were in CKD stage 3a, 8 patients were in stage 3b, 77 patients were in stage 4, 24 patients were in stage 5. Of note is that patients were recruited from our uremia list to which they were referred according to their eGFR. Once they joined the study GFR was measured and we found that eGFR often underestimated mGFR, thus explaining the discrepancy between the inclusion criteria: eGFR< 30 ml/min/1.73m^2^ and the actual results with mGFR. The CONSORT Flow Diagram is presented in Fig. 1. Clinical characteristics are presented in Table 1. The causes of CKD were hypertensive kidney disease, 47(42%), diabetic nephropathy, 21(19%), interstitial nephritis, 18(17%), chronic glomerulonephritis, 12(11%), polycystic kidney disease. In our previous RENEXC studies, we showed that physical performance, which is the primary outcome of RENEXC, tested by 6-min walking test, quadriceps strength, 30 s Sit to Stand and functional reach, improved significantly after 12 months of exercise in both groups [12, 20]. The patients in the balance group achieved a median duration of exercise of 118 [interquartile range: 64–161] minutes/week and in the strength group of 100 [interquartile range 64–187] minutes/week.

**Table 1:** Some clinical characteristics at baseline

Characteristics	Balance Group (*n* = 59)	Strength Group (*n* = 53)	Whole Group (*n* = 112)
Age, years	66 ± 13	67 ± 14	67 ± 13
Male/Female, n (%)	38 (64)/21 (36)	38 (72)/15(28)	76(68)/36(32)
Weight, kg	79 ± 16	84 ± 19	81 ± 17
Height, m	1.71 ± 0.09	1.72 ± 0.1	1.72 ± 0.09
BMI, kg/m^2^	27 ± 4.7	28.0 ± 5.2	27.4 ± 4.9
mGFR, mL/min/1.73m^2^	22 ± 7	23 ± 9	23 ± 8
P-creatinine, μmol/L	242 ± 90	251 ± 92	247 ± 91
P-urea, mmol/L	16 ± 5	15 ± 5	15 ± 5
P-PTH, pmol/L	11 (9–17)	10 (7–16)	11(8–17)
P-Albumin, g/L	37 ± 4	38 ± 3	37 ± 3
B-Hemoglobin, g/L	129 ± 14	128 ± 14	128 ± 14
P-Potassium, mmol/L	4.3 ± 0.6	4.1 ± 0.5	4.2 ± 0.5
P-Calcium, mmol/L	2.3 ± 0.1	2.3 ± 0.1	2.3 ± 0.1
P-Ca × P, mmol^2^/L^2^	2.6 ± 0.7	2.5 ± 0.5	2.6 ± 0.6
P-Phosphate, mmol/L	1.2 ± 0.2	1.1 ± 0.2	1.1 ± 0.2
Base excess, mmol /L	−1.2 (−2.3–0.1)	−1.4 (− 3.2–0.0)	−1.2 (− 2.8–0.1)
P-CRP, mg/L	2.9 (1.3–5.9)	3.5 (1.5–6.9)	3.1 (1.3–6.1)
**Medication**, n (%)			
Antihypertensive medication	55(93)	50(94)	105(94)
Calcium channel blocker	33((56)	32(60)	65(58)
Beta blocker	36(61)	37(70)	73(65)
RAAS-blocker	37(63)	34(64)	71(63)
Central antiadrenergic medication	8(14)	5(9)	13(12)
Active vitamin D	38(64)	32(60)	70(62)
Phosphate binder	24(41)	18(34)	42(38)
Calcimimetic	1(2)	1(2)	2(2)
Statin	33(56)	28(53)	61(54)
**Comorbidity**, n (%)			
Malignancy	8(14)	8(15)	16(14)
Ischemic heart disease	11(19)	11(20)	22(20)
Peripheral vascular disease	10(17)	13(25)	23(21)
Left ventricular dysfunction	8(14)	3(6)	11(10)
Diabetes mellitus	13(22)	17(32)	30(27)
Systemic collagen vascular disease	5(8)	5(9)	10(9)
Others (e.g. hypertension)	43(73)	40(75)	83(74)

Data presented as mean ± SD or median (25th–75th percentile) or n (%)

*P* Plasma, *B* Blood, *PTH* Parathyroid hormone, *Ca×P* Calcium phosphate product, *CRP* C-reactive protein, *RAAS* Renin - angiotensin - aldosterone system

### AAC score, mGFR, blood pressure and some markers of arteriosclerosis (Table 2)

After 12 months of exercise training, the AAC score increased by 1 point each in the balance group (*p* < 0.001) and in the strength group (*p* = 0.002), respectively. mGFR decreased by 1.2 mL/min/1.73m^2^ (*p* = 0.003) in the balance group and by 1.0 mL/min/1.73m^2^ (*p* = 0.01) in the strength group. Plasma lipoprotein (a) decreased by 54 nmol/L (*p* < 0.001) in the balance group and by 31 nmol/L (*p* = 0.04) in the strength group. Plasma PTH increased by 1 pmol/L(p=0.03) and 1,25(OH)2D3 increased by 5 nmol/L (P=0.04) in the strength group and were both unchanged in the balance group. Fetuin-A increased by 0.1 g/L (*p* = 0.02) in the balance group and was unchanged in the strength group. Blood pressure, triglycerides, total cholesterol, high-density lipoprotein- (HDL-C), low-density lipoprotein cholesterol (LDL-C), FGF23, phosphate, calcium, IL6, CRP and albumin were all unchanged. There was no between group difference for AAC or any of the markers measured. There was no difference in AAC score between completers (5 [0–12.5]) and non-completers (6 [0–14]).

**Table 2:** AAC score, mGFR and some markers of arteriosclerosis at baseline and after 12 months of exercise training

	Balance Group			Strength Group			Whole Group		
	Baseline	12 months	***P***	Baseline	12 months	***P***	Baseline	12 months	***P***
**AAC,** score	8.5(0–13)	9.5(1–14)	**< 0.001**	5(1–12)	6(2–13)	**0.002**	5(0–12.5)	7(2–14)	**< 0.001**
**mGFR,** mL/min/1.73m^2^	22.3 ± 6.9	21.1 ± 7.8	**0.003**	23.0 ± 9.1	22.0 ± 9.7	**0.01**	22.6 ± 8.0	21.6 ± 8.8	**< 0.001**
**Blood pressure,** mmHg	129 ± 17/75 ± 10	132 ± 17/75 ± 10	0.1	129 ± 15/74 ± 9	129 ± 13/74 ± 8	0.8	129 ± 16/ 75 ± 10	130 ± 15/ 75 ± 9	0.4
**Lipids and lipoproteins**									
P-Triglycerides, mmol/mL	1.8 ± 1.0	1.8 ± 1.0	0.7	1.8 ± 1.0	1.8 ± 1.1	0.8	1.8 ± 1.0	1.8 ± 1.0	0.2
P-Total cholesterol, mmol/L	4.8 ± 1.2	4.6 ± 1.1	0.05	4.7 ± 1.3	4.7 ± 1.3	0.6	4.8 ± 1.2	4.7 ± 1.2	0.5
P-HDL-C, mmol/L	1.3 ± 0.5	1.3 ± 0.4	0.5	1.2 ± 0.3	1.2 ± 0.4	0.6	1.3 ± 0.4	1.3 ± 0.4	0.2
P-LDL-C, mmol/L	3.0 ± 1.0	3.1 ± 2.4	0.5	2.9 ± 1.1	3.0 ± 1.1	0.5	2.9 ± 1.0	3.1 ± 1.9	0.7
Lipoprotein (a), nmol/L	99(41–209)	45(22–147)	**< 0.001**	90(29–315)	59(13–242)	**0.04**	96(31–238)	47(19–188)	**< 0.001**
**Pro-calcific markers**									
P-FGF23, ng/mL	1.8(0.6–9.5)	2.8(0.6–12.3)	0.4	1.8(0.4–6.8)	3.2(1.3–13.0)	0.4	1.8(0.5–9.2)	3.1(0.7–14.1)	0.2
P-Phosphate, mmol/L	1.2 ± 0.2	1.2 ± 0.2	0.4	1.1 ± 0.2	1.1 ± 0.3	0.2	1.1 ± 0.2	1.2 ± 0.2	0.06
P-Calcium, mmol/L	2.3 ± 0.1	2.3 ± 0.1	0.6	2.3 ± 0.1	2.3 ± 0.1	1	2.3 ± 0.1	2.3 ± 0.1	0.7
P-PTH, pmol/L	11(9.3–17)	14(8.7–20)	0.2	10(7.4–16)	11(7.7–21)	**0.03**	11(8.1–17)	13(7.9–20)	**0.01**
**Anti-calcific markers**									
P-1,25(OH)_2_D_3_, nmol/L	67 ± 31	73 ± 31	0.2	62 ± 23	67 ± 27	**0.04**	64 ± 27	70 ± 29	**0.04**
P-Fetuin-A, g/L	0.9 ± 0.3	1.0 ± 0.2	**0.02**	1.0 ± 0.3	1.0 ± 0.2	0.9	1.0 ± 0.3	1.0 ± 0.2	0.2
**Inflammatory markers**									
P-IL6, pg/mL	1.7(1–2.8)	2.1(1–3.1)	0.2	2.3(1.6–4)	2.2(1.5–3.7)	0.6	2.0(1.2–3.3)	2.1(1.3–3.3)	0.2
P-CRP (mg/L)	2.9(1.3–5.9)	3(1.2–5)	0.9	3.5(1.5–5.9)	3(1.5–6)	0.8	3.1(1.4–6.2)	3(1.4–6)	0.8
P-Albumin (g/L)	37.7 ± 2.8	36.8 ± 5.3	0.3	36.8 ± 3.6	36.0 ± 3.3	0.1	37.3 ± 3.2	36.4 ± 4.5	0.05

Data presented as mean ± SD or median(25th–75th percentile)

*AAC* Abdominal aortic calcification, *mGFR* Measured Glomerular Filtration Rate, *P* Plasma, *FGF23* Fibroblast growth factor 23, *PTH* Parathyroid hormone, *IL6* Interleukin 6, *CRP* C-reactive protein, *HDL-C* High-density lipoprotein cholesterol, *LDL-C* Low-density lipoprotein cholesterol, *95%CI* 95% confidence interval

### Relationships between delta AAC score, delta lipoprotein (a) and duration of exercise training (Tables 3 and 4)

Neither the change in AAC score nor the change in plasma lipoprotein (a) was related to the weekly duration of exercise training.

**Table 3:** Relationships between delta AAC score, delta Lipoprotein (a) and exercise duration in the balance group

	Delta AAC, Score			Delta Lipoprotein (a), nmol/mL		
	Eff ± SE	*P*	95%CI	Eff ± SE	*P*	95%CI
**Balance training duration, 10 Minutes/week increase**	0.006 ± 0.04	0.9	−0.07-0.08	2.98 ± 4.39	0.5	−5.86-11.81
**Endurance training duration, 10 Minutes/week increase**	0.002 ± 0.01	0.8	−0.03-0.02	0.39 ± 1.41	0.8	−3.21-2.44
**Total duration, 10 Minutes/week increase**	−0.003 ± 0.007	0.7	−0.02-0.01	− 0.23 ± 0.79	0.8	− 1.83-1.37

*AAC* Abdominal aortic calcification, *Eff* Efficiency, *SE* Standard error, *95%CI* 95% confidence interval

**Table 4:** Relationships between delta AAC score, delta Lipoprotein (a) and exercise duration in the strength group

	Delta AAC, Score			Delta Lipoprotein (a), nmol/mL		
	Eff ± SE	*P*	95%CI	Eff ± SE	*P*	95%CI
**Strength training duration, 10 Minutes/week increase**	0.02 ± 0.04	0.6	−0.10-0.06	−3.57 ± 3.92	0.4	−11.50-4.36
**Endurance training duration, 10 Minutes/week increase**	− 0.02 ± 0.02	0.4	− 0.06-0.03	−1.11 ± 2.13	0.6	−5.43-3.21
**Total training duration, 10 Minutes/week increase**	− 0.01 ± 0.02	0.4	−0.05-0.02	− 0.94 ± 1.55	0.5	−4.08-2.19

*AAC* Abdominal aortic calcification, *Eff* Efficiency, *SE* Standard error, *95%CI* 95% confidence interval

### Relationships between delta AAC score, mGFR and some markers of arteriosclerosis

After adjusting for sex, age and mGFR, delta AAC score showed a positive significant relationship with baseline plasma triglycerides (*p* = 0.01). Delta AAC was not significantly related to any other measured baseline markers. Age, however, emerged as a determinant of AAC: delta AAC was significantly related to a 1-year increase in age (*p* < 0.01) (Table 5).

**Table 5:** Relationships between delta AAC score and some markers of arteriosclerosis at baseline after adjusting for age, sex and mGFR

Delta AAC Score (12 month minus baseline)			
	Eff ± SE	*P*	95%CI
**mGFR,** 1 ml/min/1.73m^2^	− 0.02 ± 0.01	0.2	− 0.04-0.01
Age, 1-year increase	0.02 ± 0.01	0.01	0.005–0.04
Sex, Male	−0.12 ± 0.23	0.6	−0.58-0.34
AAC, Score	− 0.01 ± 0.02	0.6	−0.05-0.03
Age, 1-year increase	0.02 ± 0.01	0.02	0.004–0.05
Sex, Male	−0.15 ± 0.24	0.5	−0.63-0.33
mGFR, 1 mL/min/1.73m^2^ increase	−0.02 ± 0.01	0.2	−0.05-0.01
**Lipids and lipoproteins**			
P-Triglycerides, mmol/mL	0.28 ± 0.11	**0.01**	0.07–0.49
Age, 1-year increase	0.03 ± 0.01	0.003	0.01–0.04
Sex, Male	−0.12 ± 0.23	0.6	−0.57-0.34
mGFR, 1 mL/min/1.73m^2^ increase	−0.02 ± 0.01	0.2	−0.04-0.01
P-Total cholesterol, mmol/mL	0.16 ± 0.09	0.1	−0.03-0.35
Age, 1-year increase	0.02 ± 0.01	0.01	0.01–0.04
Sex, Male	− 0.05 ± 0.24	0.9	−0.53-0.44
mGFR, 1 mL/min/1.73m^2^ increase	−0.02 ± 0.01	0.2	−0.04-0.01
HDL-C, mmol/mL	−0.29 ± 0.30	0.3	−0.88-0.30
Age, 1-year increase	0.02 ± 0.01	0.01	0.005–0.04
Sex, Male	−0.22 ± 0.26	0.4	−0.73-0.29
mGFR, 1 mL/min/1.73m^2^ increase	−0.02 ± 0.01	0.3	−0.04-0.01
LDL-C, mmol/mL	0.15 ± 0.11	0.2	−0.06-0.36
Age, 1-year increase	0.02 ± 0.01	0.01	0.01–0.04
Sex, Male	−0.08 ± 0.24	0.7	−0.55-0.39
mGFR, 1 mL/min/1.73m^2^ increase	−0.02 ± 0.01	0.2	−0.04-0.01
Lipoprotein (a), nmol/mL	0.001 ± 0.0004	0.1	−0.0001-0.001
Age, 1-year increase	0.02 ± 0.01	0.03	0.002–0.04
Sex, Male	0.02 ± 0.25	0.9	−0.47-0.50
mGFR, 1 mL/min/1.73m^2^ increase	−0.01 ± 0.01	0.3	−0.04-0.01
**Calcific markers**			
P- FGF23, ng/mL	0.0001 ± 0.0009	0.9	−0.002-0.002
Age, 1-year increase	0.02 ± 0.009	0.01	0.005–0.04
Sex, Male	0.02 ± 0.25	0.9	−0.52-0.47
mGFR, 1 mL/min/1.73m^2^ increase	−0.01 ± 0.01	0.3	−0.04-0.01
P-Phosphate, mmol/L	−0.69 ± 0.55	0.2	−1.79-0.41
Age, 1-year increase	0.02 ± 0.01	0.01	0.005–0.04
Sex, Male	−0.19 ± 0.24	0.4	−0.66-0.29
mGFR, 1 mL/min/1.73m^2^ increase	−0.03 ± 0.02	0.1	−0.06-0.01
P-Calcium, mmol/L	1.10 ± 0.99	0.3	−0.86-3.06
Age, 1-year increase	0.02 ± 0.01	0.01	0.005–0.04
Sex, Male	−0.10 ± 0.24	0.7	−0.58-0.38
mGFR, 1 mL/min/1.73m^2^ increase	−0.02 ± 0.01	0.2	−0.04-0.01
P-PTH, pmol/L	−0.006 ± 0.01	0.3	−0.02-0.01
Age, 1-year increase	0.02 ± 0.01	0.01	0.004–0.04
Sex, Male	−0.12 ± 0.24	0.6	−0.59-0.36
mGFR, 1 mL/min/1.73m^2^ increase	−0.02 ± 0.01	0.1	−0.05 − 0.01
P-1,25(OH)_2_D_3_, nmol/L	0.003 ± 0.004	0.7	-0.01-0.01
Age, 1-year increase	0.02 ± 0.01	0.02	0.004–0.04
Sex, Male	−0.17 ± 0.25	0.5	−0.66-0.32
mGFR, 1 mL/min/1.73m^2^ increase	−0.01 ± 0.02	0.5	−0.04-0.02
P- Fetuin-A, g/L	−0.21 ± 0.33	0.5	−0.87-0.45
Age, 1-year increase	0.02 ± 0.01	0.02	0.004–0.04
Sex, Male	−0.13 ± 0.24	0.6	−0.60-0.34
mGFR, 1 mL/min/1.73m^2^ increase	−0.01 ± 0.01	0.3	−0.04 − 0.01
**Inflammatory markers**			
P-IL6, pg/mL	0.06 ± 0.03	0.09	-0.01-0.12
Age, 1-year increase	0.02 ± 0.01	0.02	0.004–0.04
Sex, Male	−0.17 ± 0.23	0.5	−0.64-0.29
mGFR, 1 mL/min/1.73m^2^ increase	−0.01 ± 0.01	0.3	−0.04-0.01
P-CRP, mg/L	0.01 ± 0.02	0.7	−0.03-0.04
Age, 1-year increase	0.02 ± 0.01	0.02	0.004–0.04
Sex, Male	−0.11 ± 0.24	0.6	−0.58-0.36
mGFR, 1 mL/min/1.73m^2^ increase	−0.02 ± 0.01	0.3	−0.04-0.01
P-Albumin, g/L	0.004 ± 0.03	0.9	−0.06-0.07
Age, 1-year increase	0.02 ± 0.01	0.01	0.005–0.04
Sex, Male	−0.12 ± 0.23	0.6	−0.59-0.34
mGFR, 1 mL/min/1.73m^2^ increase	−0.02 ± 0.01	0.3	−0.04-0.01

*AAC* Abdominal aortic calcification, *mGFR* Measured Glomerular Filtration Rate, *P* Plasma, *FGF23* Fibroblast growth factor 23, *PTH* Parathyroid hormone, *IL6* Interleukin 6, *CRP* C-reactive protein, *HDL-C* High-density lipoprotein cholesterol, *LDL-C* Low-density lipoprotein cholesterol, *95%CI* 95% confidence interval, *Eff* Efficiency, *SE* Standard error

We also analyzed the threshold effects by comparing delta AAC score at lower and upper quartiles of each marker in the whole group. Patients with higher baseline levels (upper quartile) of lipoprotein (a) had a higher increase in AAC score (0.5 [0–1.3]) than those with lower levels (lower quartile) of lipoprotein (a) (0 [0–1]), (*p* = 0.03). No significance was found for any other markers.

## Discussion

After 12 months of either balance- or strength- in combination with endurance training, both groups improved physical performance significantly; showed significant increases in AAC score; significant decreases in mGFR and lipoprotein (a). PTH and 1,25(OH)_2_D_3_ increased significantly in the strength group only; fetuin-A increased significantly in the balance group only. Triglycerides, total cholesterol, HDL-C and LDL-C, plasma FGF23, phosphate, calcium, IL6, CRP, albumin were all unchanged in both groups. There were no between group differences for AAC or any of the markers. The increase in AAC score was positively related to ageing and to the levels of plasma triglycerides at baseline. Neither the increase in AAC score nor the decrease in plasma lipoprotein (a) was related to the exercise dose, measured as weekly duration of exercise training.

High levels of plasma lipids and lipoproteins have been observed in atherosclerotic lesions and are considered risk factors for atherosclerosis [21]. Earlier studies have shown that exercise training is effective in reducing the levels of plasma triglycerides, LDL-C, while simultaneously increasing the levels of plasma HDL-C [22, 23]. But we did not find any changes in plasma triglycerides, LDL-C or HDL-C in this study. However, no study, to our knowledge, has shown that exercise training could reduce lipoprotein (a) [24, 25]. Lipoprotein (a) is a lipoprotein variant, and has been found as an intact particle in the arterial intimal layer, particularly in association with atherosclerotic plaques [26]. In this study, a 51% decrease in lipoprotein (a) was observed after 12 months of exercise training. The decrease was more significant in the balance group, but without a between group difference; the decrease in lipoprotein (a) was not related to the weekly exercise dose. This decrease, in lipoprotein (a) in our study, could well convey a positive effect of exercise training on the intimal vascular layer [2].

In CKD, the dysregulation of the FGF23-klotho endocrine axis is suggested to be one of the underlying mechanisms leading to vascular calcification [3]. The levels of FGF23 are around 0.03 ng/mL in healthy subjects [17], start increasing during CKD stage 2, and reach levels more than 1000-fold above normal in patients on dialysis [17]. In our group of patients with CKD stages 3–5, the levels of plasma FGF23 were elevated around 100-fold above values in healthy subjects [17] at baseline and remained stable after 12 months of exercise training, despite a significant, albeit modest, decrease in GFR. Despite stable plasma calcium, phosphate and FGF23 levels and a significant increase in 1,25(OH)_2_D_3_ in the strength group, there was an increase in PTH in the strength group after 12 months, which was not related to baseline mGFR. A possible explanation for the rise in PTH could be the contribution of PTH to bone adaptation during exercise, as shown in mice and healthy human subjects [27, 28].

Fetuin-A is an extracellular calcium-regulatory protein, which acts as a strong inhibitor of calcium - phosphate deposition and an inhibitor of vascular calcification [1]. Fetuin-A increased in the balance group after 12 months of exercise training.

Inflammation is a known promoter of vascular calcification. It not only induces damage to vascular smooth muscle cells, but can also cause a decrease in hepatic secretion of fetuin-A [4]. Other studies have suggested that exercise training could reduce the plasma levels of CRP and IL6 in patients with non-dialysis dependent CKD [10, 29] . However, in one report in patients on dialysis, neither IL6 nor CRP changed after exercise training [30]. Plasma albumin did not change in any of these three above mentioned studies [10, 29, 30]. We found, in our larger sample of patients, with a longer period of intervention, that plasma IL6, CRP and albumin remained within normal ranges and were unchanged after 12 months of exercise training. In consequence, the effects of exercise training on systemic inflammation in patients with CKD are far from clear and it is possible that plasma inflammatory markers do not provide the full picture.

A recent longitudinal study, in highly active middle-aged recreational athletes, showed that progression of coronary artery calcification was not associated with the volume of endurance training, but was associated with age and baseline coronary artery calcification [31]. Our results are similar insofar, as 12 months of regular low to moderate intensity exercise training in patients with CKD did not halt the progression of AAC, despite well controlled calcific- and inflammatory markers and significant improvements in walking distance, quadriceps strength, muscular endurance in the legs and balance. Although, we did not find any relationship between baseline AAC and progression of AAC. Vascular calcification, induced by a declining GFR, accumulates over a long period of time so 12 months of low to moderate intensity exercise training might be too brief a period and/or too weak a stimulus to halt or reverse this process. As GFR declines, FGF23 and other pro-calcific and inflammatory markers increase [17, 32], anti-calcific markers, like fetuin-A and 1,25(OH)_2_D_3_, decrease [18, 33]. All these changes are associated with higher risks of morbidity and mortality [1, 18, 33, 34]. It is noteworthy, that although exercise training did not have an impact on AAC per se; FGF23, phosphate and calcium were maintained despite a modest decrease in mGFR; lipoprotein (a) decreased in both groups and fetuin-A increased in the balance group. One could speculate that the balance exercises involve larger muscle groups than strength exercises, which might have had an impact.

As FGF23 starts increasing during CKD stage 2 [4, 17], exercise training initiated earlier might have had a stronger preventative effect on the development of vascular calcification, especially as most of our patients were in CKD stages 4 and 5. In a previous cross-sectional study, we showed that the AAC score was associated with a decline in GFR and an increase in plasma phosphate [35], but causal relationships were not found in the present longitudinal study. Nor did we find any causal relationships between AAC score and any of the other measured calcific- and inflammatory markers. However, we found that the progress in AAC was positively related to the levels of baseline plasma triglycerides, which suggests that hypertriglyceridemia might contribute to arteriosclerosis in this group of patients. The increase in AAC score also showed a threshold relationship with lipoprotein (a) at baseline, though a linear relationship was not found. Patients with higher baseline levels of lipoprotein (a) showed a greater progression of AAC than patients with lower baseline levels. Additionally, the levels of lipoprotein (a) decreased significantly after exercise as mentioned above. Consequently, exercise might affect AAC by reducing the levels of lipoprotein (a). High levels of LDL-C have previously been reported to promote the progress of vascular calcification [36, 37], but this result was not confirmed in our study. Of special note is that age had a highly significant longitudinal relationship with the AAC score, which means that ageing was one of the strongest drivers of vascular calcification. In a recent study, AAC volume was evaluated after on average 3.4 years in patients with CKD stages 3 to 4 and an average mGFR of 37 ml/min/1.73m^2^, the authors found that age was the only statistically independent predictor of AAC [38].

This study has some strengths. Firstly, this group of patients is representative of typical non-dialysis dependent patients with CKD in the later stages, as the majority are elderly and suffer from a number of comorbidities. Secondly, the patients have well-controlled levels of phosphate, calcium and PTH. Thirdly, this is a longitudinal study in which patients were followed for 12 months. This study also has some limitations. Firstly, there was no sedentary control group. Secondly, the majority of our patients were at CKD stages 4 and 5 at which the deranged metabolic axis of calcium-phosphate-PTH/FGF23 is well established as are the atherosclerotic changes driven by the progression of uremia. In consequence, our patients presented with quite an advanced degree of aortic calcification at baseline making it less likely that exercise training would affect its development. In this context it would be interesting to study the effects of exercise training in patients at earlier CKD stages and compare them with sedentary controls. Thirdly, due to methodological difficulties, we were not able to assay plasma klotho so that we could not follow changes in the whole FGF23-klotho axis after exercise training.

## Conclusion

In conclusion, in these non-dialysis dependent patients with CKD stages 3–5, 12 months of exercise training did not prevent the progression of AAC. It might have contributed to the reduction of lipoprotein (a) levels and the maintenance of the concentrations of pro-calcific- and anti-inflammatory markers. Hypertriglyceridemia, high levels of lipoprotein (a) and ageing emerged as longitudinal predictors of vascular calcification in these patients. Further studies on the progression of AAC during the natural course of CKD and after exercise are required.

## Data Availability

The datasets used and/or analysed during the current study are available from the corresponding author on reasonable request.

## Abbreviations

AAC: Abdominal aortic calcification
CKD: Chronic kidney disease
CRP: C-reactive protein
ELISA: Enzyme-linked immunosorbent assay
FGF23: Fibroblast growth factor 23
GFR: Glomerular filtration rate
eGFR: estimated GFR
mGFR: measured glomerular filtration rate
HDL-C: High density lipoprotein cholesterol
IL6: Interleukin 6
KDIGO: Kidney Disease Improvement Global Outcomes
LDL-C: Low density lipoprotein cholesterol
MGP: Matrix Gla protein (MGP)
PTH: Parathyroid hormone
RENEXC: Renal exercise
SWEDAC: Swedish Board for Accreditation and Conformity Assessment
